# Redshifted and Near‐infrared Active Analog Pigments Based upon Archaerhodopsin‐3

**DOI:** 10.1111/php.13093

**Published:** 2019-04-08

**Authors:** Srividya Ganapathy, Svenja Kratz, Que Chen, Klaas J. Hellingwerf, Huub J.M. de Groot, Kenneth J. Rothschild, Willem J. de Grip

**Affiliations:** ^1^ Leiden Institute of Chemistry Leiden University Leiden The Netherlands; ^2^ Swammerdam Institute for Life Sciences University of Amsterdam Amsterdam The Netherlands; ^3^ Molecular Biophysics Laboratory Photonics Center and Department of Physics Boston University Boston MA; ^4^ Department of Biochemistry Radboud University Medical Center Nijmegen The Netherlands

## Abstract

Archaerhodopsin‐3 (AR3) is a member of the microbial rhodopsin family of hepta‐helical transmembrane proteins, containing a covalently bound molecule of all‐*trans* retinal as a chromophore. It displays an absorbance band in the visible region of the solar spectrum (*λ*max 556 nm) and functions as a light‐driven proton pump in the archaeon *Halorubrum sodomense*. AR3 and its mutants are widely used in neuroscience as optogenetic neural silencers and in particular as fluorescent indicators of transmembrane potential. In this study, we investigated the effect of analogs of the native ligand all‐*trans* retinal A1 on the spectral properties and proton‐pumping activity of AR3 and its single mutant AR3 (F229S). While, surprisingly, the 3‐methoxyretinal A2 analog did not redshift the absorbance maximum of AR3, the analogs retinal A2 and 3‐methylamino‐16‐nor‐1,2,3,4‐didehydroretinal (MMAR) did generate active redshifted AR3 pigments. The MMAR analog pigments could even be activated by near‐infrared light. Furthermore, the MMAR pigments showed strongly enhanced fluorescence with an emission band in the near‐infrared peaking around 815 nm. We anticipate that the AR3 pigments generated in this study have widespread potential for near‐infrared exploitation as fluorescent voltage‐gated sensors in optogenetics and artificial leafs and as proton pumps in bioenergy‐based applications.

## Graphical abstract

The proton pump archaerhodopsin‐3 (AR3) is widely used in optogenetics as a silencer of neural activity or as a fluorescent indicator of transmembrane potential. In this research, we utilize analogs of its native chromophore all‐*trans* retinal to generate variants of AR3 and a single mutant. One analog from this study generated redshifted AR3 pigments which could be activated by near‐infrared light. Furthermore, these pigments showed strongly enhanced fluorescence with an emission band in the near‐infrared. We anticipate that these analog AR3 pigments have potential for exploiting near‐infrared light in several optogenetic and bioenergy‐based applications.

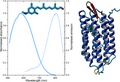

## Introduction

Microbial rhodopsins are a family of hepta‐helical transmembrane proteins found in a diverse array of micro‐organisms spanning archaea, bacteria and eukaryotes. They function as light‐driven ion pumps, channels or sensors and play a vital role in survival and adaptation of their host organisms 1, 2. Due to their diverse functions and ability to tune the spectral properties of their chromophore all‐*trans* retinal, microbial rhodopsins have been utilized in important biotechnological applications. For instance, light‐driven rhodopsin proton pumps have phototrophic potential complementing natural photosynthesis 3, 4, 5 or in biomolecular nanodevices 6. Furthermore, light‐gated ion channels are expressed in neurons and used to selectively depolarize or hyperpolarize cells upon illumination in the field of optogenetics 7. This enables precise spatiotemporal control of neuronal activity.

Bacteriorhodopsin (BR) was the first microbial rhodopsin to be discovered from the archaeon *Halobium salinarum* in 1971 8. The photocycle, 3‐D structure and light‐driven structural changes of BR have been investigated in considerable detail, making BR the gold standard to compare the properties of other microbial rhodopsins 9. Since the discovery of BR, several more ion‐pumping rhodopsins have been found in archaea, of which the archaerhodopsins are most notable. Archaerhodopsin‐1 and archaerhodopsin‐2 (AR1 and AR2) were discovered in *Halorubrum sodomense* 10, and their crystal structures have since been resolved 10, 11. They, like BR, function as retinal containing light‐driven proton pumps, sharing about 75% homology with BR 12, 13.

Considerable interest has also focused recently on the variant archaerhodopsin‐3 (AR3) found in *H. sodomense*, due to its application as a high‐performance light‐triggered neural silencer in optogenetics 14. AR3 and its nonpumping mutant AR3 (D95N) are also utilized as sensors of transmembrane potential, due to their voltage‐gated fluorescence 15. The first fluorescent membrane sensor was derived from the eubacterial proteorhodopsin (PR) and dubbed proteorhodopsin optical proton sensor (PROPS) 16. PROPS has been expressed and extensively characterized in *Escherichia coli (E. coli)* 16. However, it was observed that in mammalian cells, PROPS was difficult to express and target to the cellular membrane, which strongly limits its applicability in optogenetics. On the other hand, AR3 shows excellent mammalian expression and targeting 14 and has been widely used in the investigation of a variety of neural and cardiac systems 17, 18.

Like all microbial rhodopsins, AR3 covalently binds a molecule of all‐*trans* retinal via a Schiff base (SB) linkage with a lysine residue (Lys226). In the ground state, the SB is protonated (PSB) and this positive charge is balanced by a negatively charged “soft counterion” consisting of two Asp residues (Asp95 and Asp212) and water molecules 10, 19, 20. Overall, the retinal chromophore structure and Schiff base interactions of AR3 are very similar to BR 20. Upon absorbing a photon, the all‐*trans* chromophore isomerizes to the 13‐*cis* configuration triggering proton transfer to the primary proton acceptor Asp95. Ultimately, for each isomerization event, one proton is transported across the cell membrane due to proton transfers involving both a network of strongly hydrogen‐bonded water molecules and hydrogen‐bonding protein residues, which are conserved between BR and AR3 21.

**Figure 1:**
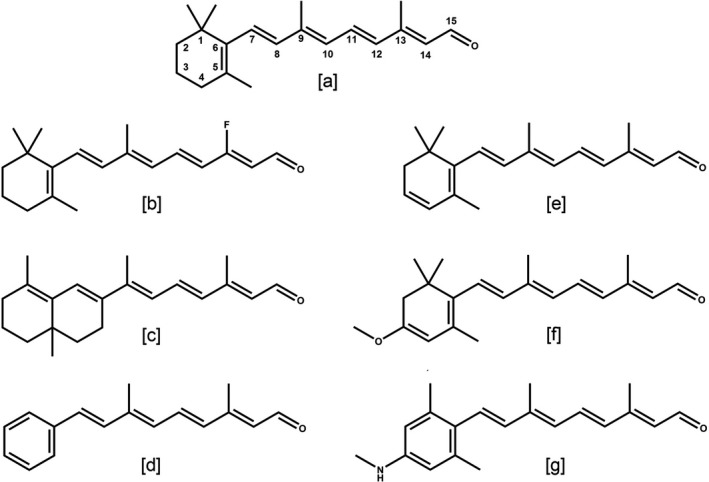
Chemical structures of retinal analogs tested in this study: (a) A1, the native retinal (b) 14F, (c) ALL‐E, (d) PHE, (e) A2, (f) MOA2 and (g) MMAR. Official nomenclature and spectral properties are detailed in the Fig. S1.

Due to the sensitivity of the retinal chromophore to the structural constraints and the local electrostatic environment imposed by the retinal binding pocket, chromophore modification in combination with mutagenesis has been used successfully to spectrally tune microbial rhodopsins 22, 23, 24. For instance, the spectral profile of AR3 has been redshifted using retinal A2 (A2 in Fig. 1) without a significant effect on the charge transport and photocycle kinetics, as measured via the changes in the light‐induced transmembrane electrical potential 25. In view of its application as a fluorescent voltage sensor, several variants of AR3 have been engineered to achieve an up to eight‐fold increase in brightness, using retinal analogs 26, site‐saturation mutagenesis 27 and a combination of both 28, 29. In another approach, incorporation of conjugated‐chain extended retinals into AR3 induced a switch of function from light‐driven proton pump to light‐driven proton channel 30.

The amalgamation of retinal analogs with protein modification has proven to be an effective strategy toward spectrally tuning microbial rhodopsins. For instance, we previously showed that retinal analogs in combination with mutagenesis can modulate both the spectral properties and the pump activity of PR and *Gloeobacter violaceus* rhodopsin (GR) 22, 23. This i.a. resulted in analog pigments active in near‐infrared (NIR) light.

In this study, we test several retinal analogs (Fig. 1) on the spectral properties and proton pump function of AR3 and its single mutant AR3(F229S). Most of these analogs have not been tested before on archaeal opsins. The novel F229S mutation induces a small redshift and improves expression level and pumping rate of AR3. We show that for AR3 and AR3(F229S) the selectivity for and interaction with the tested retinal analogs differ from the eubacterial PR and GR. Another important result was that incorporation of the MMAR analog in AR3 and AR3 (F229S) yielded NIR‐active proton pumps as well as strong NIR‐activated fluorescence emission.

## Materials and Methods

### Materials

All‐*trans* retinal (A1) was obtained from Sigma‐Aldrich. All‐*trans*‐3,4‐dehydroretinal (A2), all‐*trans* all‐*E*‐locked retinal (ALL‐E) and all‐*trans* 14‐fluoro‐retinal (14F) were generous gifts from Hoffman‐LaRoche, Prof. Johan Lugtenburg 31 and Prof. Robert Liu 32, respectively. All‐*trans* 3‐methoxy‐3,4‐dehydroretinal (MOA2) and all‐*trans* 3‐methylamino‐16‐nor‐1,2,3,4‐didehydroretinal (MMAR) were synthesized on order from Buchem BV (Apeldoorn, The Netherlands). Sources of chemicals include the following: isopropyl *β*‐d‐1‐thiogalactopyranoside (IPTG; Promega), 1‐n‐dodecyl‐*β*‐d‐maltopyranoside (DDM, Protein Labelling Innovation), benzonase (Novagen), lysozyme (Sigma) and Ni^2+^‐NTA columns (Thermo Scientific). All other chemicals were of the highest purity, available.

### Plasmids and cell lines used

*Escherichia coli* strain BL21DE3 was transformed with the pet28b plasmid containing a cassette for kanamycin resistance and encoding for the IPTG inducible AR3 gene (UniProtKB: P96787) with a 6xHis tag on the C‐terminal. Codon usage was optimized for mammalian cells, which did not deter expression in *E. coli* 19.

### Site‐directed mutagenesis

The pet28b plasmid was linearized by restriction with MluI endonuclease and subjected to mismatch PCR using overlapping primers containing the corresponding mutation sites for the F229S mutation as described previously for PR and GR (see Appendix S1 for primer sequences) 22, 23. The amplified mutant gene and vector were restricted at the EcoRI and NcoI sites and run on an agarose gel. The bands were cut out, extracted and ligated overnight at 4°C. The ligated plasmid was transformed into *E. coli* BL21DE3 made chemically competent using calcium chloride 33.

### Cell culturing and regeneration

The *E. coli* cells were grown in Terrific broth (TB) medium with 100 μg mL^−1^ kanamycin at 37°C in an orbital shaker at 180 rpm. Overnight cultures were grown from frozen glycerol stocks of transformed cells, which were diluted 10 times to get the working culture. At a cell density corresponding to an OD_600_ of 0.8–0.9, expression of the holoprotein was induced by the addition of IPTG to a final concentration of 0.5 mm with concomitant addition of retinal to a final concentration of 20 μm as described previously 23. The cells were allowed to continue to grow for another 6 h in the dark.

### Protein purification

The cells were spun down and washed twice with an equal volume of 150 mm NaCl. Finally, the pellet was resuspended in ice‐cold lysis buffer (5 mL/100 mL culture volume) containing 20 mm Tris, 50 mm NaCl, 20 mm imidazole, 0.1% 1‐n‐dodecyl‐*β*‐d‐maltopyranoside (DDM), pH 7, supplemented with an EDTA‐free protease inhibitor tablet, benzonase (4 units/100 mL culture) and lysozyme (4 mg/100 mL culture). The suspension was sonicated at 4°C using a Sonics vibra‐cell sonicator (4 s on, 5 s off, 30% amplitude) and centrifuged to remove cellular debris. DDM was then added to a final concentration of 2.5% (w/v) to solubilize membrane components, and the sample was kept rotating at room temperature for a day. The insoluble material was removed by centrifugation (4000 ***g***, 25 min, 4°C). From the resulting supernatant, the C‐terminally His‐tagged AR3 was purified using immobilized‐nickel affinity chromatography (IMAC) and characterized by absorbance spectroscopy, as described previously for eubacterial rhodopsins 23.

### Proton‐pumping measurement and pigment quantification

Suspensions of *E. coli* cells expressing either AR3 or AR3(F229S), obtained from a 50 mL culture supplemented with a selected retinal (Fig. 1), were starved for four days in buffer containing 250 mm KCl, 10 mm NaCl, 10 mm MgSO_4_, 100 μm CaCl_2_, 10 mm Tris‐HCl pH 7 and subsequently directly used to measure light‐induced proton‐pumping activity as described previously for eubacterial rhodopsins 23. Further details including light sources and illumination conditions are described in the Supporting Information. After the activity measurements, the same samples were assayed for AR3 content. Hereto, the *E. coli* suspensions were harvested by centrifugation (3200 ***g***, 20 min, RT), and the pellet was resuspended in an ice‐cold solution of buffer A (50 mm Tris‐HCl, 150 mm NaCl, pH 7; 10 mL per 50 mL of culture). The suspended cells were lysed by sonication at 4°C (10 min, 4 s pulses, 5 s pauses, 25% amplitude). The resulting cellular debris and membrane vesicles were pelleted together by high‐speed centrifugation (147 000 ***g***, 1 h, 4°C). The pellet was resuspended in buffer A (2 mL per 50 mL culture), and DDM was added to a final concentration of 2.5% (w/v) followed by overnight incubation at 4°C under rotation. The insoluble material was removed by centrifugation (21 000 ***g***, 30 min, 4°C). Under these conditions, maximum extraction of all pigment species was achieved without significant losses. The resulting supernatant was used for spectral analysis upon treatment with hydroxylamine, as described below. The absorbance at the *λ*max was used to calculate the original pigment level in the cell suspension. A value of 50 300 m ^−1^ cm^−1^ was reported as the molar absorbance coefficient of AR3 34. This is in line with the values reported for BR (54 000 m ^−1^ cm^−1^ 8 and 63 000 m ^−1^ cm^−1^ 35 and for PR and GR (54 200 and 55 500 m ^−1^ cm^−1^, respectively 23). However, because of the lower stability of the AR3 analog pigments after purification, we could not reliably measure their molar absorbance coefficients. In view of the similarity in binding pocket residues for AR3 and GR (Table S1) and their congruent spectral properties, we decided to use the reported molar absorbance of GR and its analog pigments also for AR3 23. This will create a 10–20% uncertainty in the calculated molecular proton‐pumping rates, but this will not affect the general trend of the data. Hence, in Table 1, we only specify the maximal molecular rates attained under a specific illumination condition, and the other rates in that same illumination condition have been normalized to that maximal rate and categorized in a number of intervals to show the general trend.

### Absorbance spectroscopy

All absorbance spectra were measured on a Shimadzu UV‐Vis spectrophotometer (UV‐1601) at room temperature. Several AR3 analog pigments were not very stable in DDM solution at room temperature. Although the stability of these pigments could be improved by inclusion of antioxidant (1 mm DTT) and protease inhibitor (Roche) during solubilization and purification, spectra of purified pigments became contaminated with liberated retinals. In that case, the major absorbance band of the AR3 pigment was extracted from the composite spectrum of membrane vesicles. After solubilization (2.5% DDM, 4°C, 12 h), a spectrum was recorded and subsequently hydroxylamine was added from a 1 m stock solution, pH 7, to a final concentration of 200 mm, followed by incubation at RT under white light illumination (800 μmol m^−2^ s^−1^). Most pigments required up to 30 min of illumination for full bleaching, except for AR3:MMAR and AR3 (F229S):MMAR which were completely bleached within 3 min. Hydroxylamine attacks the Schiff base and releases the retinal from the opsin binding pocket as retinaloxime. The difference spectrum, obtained by subtracting the spectrum after hydroxylamine treatment from the one before, then reveals the major absorbance band of the AR3 pigment.

### Fluorescence spectroscopy

Emission spectra were recorded in 0.1–0.2% (w/v) DDM solution on a Fluoramax‐3 UV/Vis fluorescence spectrometer (HORIBA) at room temperature. The samples were excited at the *λ*max values of their absorbance band (Table 1). The slit size for excitation was adjusted to 5 nm. The excitation light intensity was monitored by a photodiode, and the fluorescence intensity was corrected for the photon flux of the excitation light. Pigment concentrations ranged from 2 to 4 μm.

### Homology modeling

A homology model for AR3 was constructed using the structure of BR as a template (PDB: 3HAR) 36, which has 57% sequence identity. Model building and subsequent analysis were performed using the WHAT IF (PMID:2268628) and YASARA (PMID:11948792) Twinset with standard parameters.

## Results and Discussion

### Regeneration and yield of AR3 and AR3(F229S)

**Figure 2:**
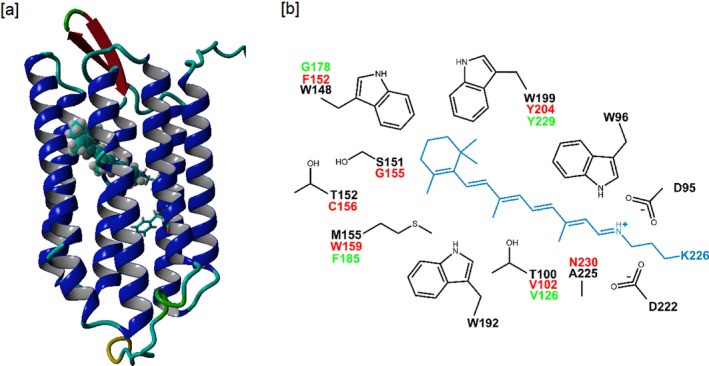
(a) Homology model of AR3, created and visualized using YASARA, as described under Materials and Methods. The mutation site F229 is highlighted in the form of a “stick model” of its side chain, while the retinal chromophore is displayed as a space filled residue in cyan. (b) Schematic of the retinal binding pocket with the retinylidene Schiff base–lysine moiety. Part of the binding pocket residues in AR3, listed in Table S1, is shown here with complete side‐chain structures. This includes all residues variant between AR3, GR and PR. Black numbered residues derive from AR3. BR has identical residues in these positions. Variant residues are shown in red for PR and in green for GR. For a complete binding pocket residue listing, see Table S1.

The point mutation F229S was generated in AR3 in analogy to the orthologous mutations F234S in PR and F260S in GR from a previous study, based upon the homology models we produced (Fig. 2) 22. The latter two mutations induced a very large redshift in the MMAR analog pigment of PR and increased pumping activity in GR, respectively 23. *E. coli* BL21DE3 was used to recombinantly express 6xHis tagged AR3 and AR3(F229S). Expression of the apoprotein (opsin) could be induced upon addition of IPTG, and the holoprotein was formed upon simultaneous addition of IPTG and retinal to the cell culture. In contrast to PR and GR, maximal regeneration of AR3 opsin into the holoprotein was only achieved upon addition of retinal to the cell culture simultaneously with induction of AR3 expression. Supplementation with retinal at a later stage resulted in much less or no holoprotein production. Holoprotein formation resulted in bright purple cell pellets. An expression level of ca 5 × 10^4^ molecules/cell was measured for AR3, while AR3 (F229S) showed an about 1.5‐fold higher expression level. These expression levels are in the same range as achieved with GR and PR, while enhanced expression was also observed for GR (F234S) (GR‐FS) relative to GR 22, 23. With the exception of PHE, all the retinal analogs tested in this study (Fig. 1) could be stably incorporated in both AR3 and AR3(F229S) and yielded excellent regeneration levels when supplemented in cell culture.

### Spectral properties of the AR3 pigments

**Figure 3:**
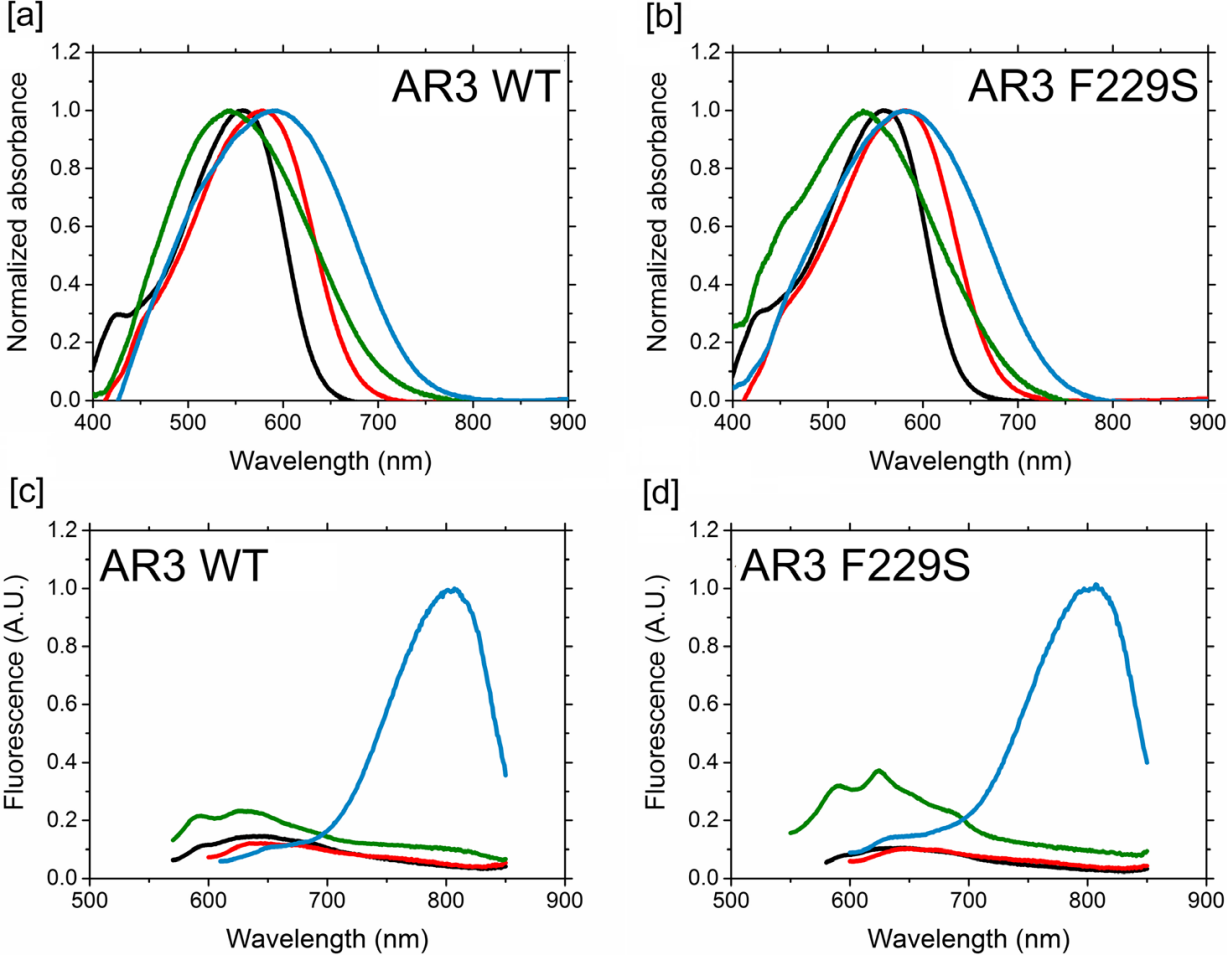
Normalized absorbance and emission spectra of retinal‐based pigments generated with AR3 (a) and AR3‐FS (b) containing A1 (black), A2 (red), MOA2 (green) or MMAR (blue). (a, b) Difference spectra obtained in 2.5% DDM solution before and after reaction with hydroxylamine. (c, d) Fluorescence emission spectra of the same pigments were obtained upon excitation at their *λ*_max_. For details, see Materials and Methods. The fluorescence intensity of the pigments was normalized against the maximal intensity of the MMAR analog, which was set at 1 A.U.

AR3 containing the native ligand retinal A1 (AR3:A1) could be purified to a high degree, exploiting the 6xHis tag and using immobilized Ni^2+^ affinity chromatography with 1‐n‐dodecyl‐*β*‐d‐maltopyranoside (DDM) as detergent (Fig. S2, see Supporting Information). Purified pigments were eluted in 0.1% DDM for optimal thermal stability. AR3:A1 showed a main absorbance band peaking at 556 nm, in agreement with previous reports for AR3 expressed in *E. coli* 25, 30. The mutation F229S caused a modest 4 nm redshift in the absorbance band of the corresponding A1 pigment AR3(F229S):A1, compared to AR3:A1 (Fig. 3, Table 1). This is again comparable to the F260S mutation in GR which only generates a 9 nm redshift, in contrast to the 20 nm redshift seen for the orthologous mutation in PR (F234S):A1.

Of the retinal analogs tested in this study (Fig. 1), AR3 and AR3(F229S) pigments containing A2 were sufficiently stable to allow purification. On the other hand, pigments containing MOA2, ALL‐E, MMAR and 14F were stable in a membrane environment, but slowly denatured during the purification procedure, even when the mild detergent DDM was employed. The main absorbance band of these pigments could therefore only be accurately recorded via rapid solubilization in 2.5% DDM at 4°C followed by bleaching of this band into the retinaloxime derivative by illumination in the presence of hydroxylamine, and calculating the difference spectrum. The main absorbance bands of all pigments generated in this manner are presented in Figs. 3 and S3, and the corresponding absorbance maxima are compiled in Table 1. The A1 and A2 pigments were stable at 4°C in 0.1% DDM solution for several days, while the other pigments showed poor thermal stability in detergent and even in 0.1% DDM required storage at −80°C. The MOA2, ALL‐E, 14F and in particular MMAR pigments are more vulnerable to destabilization by detergent, which is also reflected in the much more rapid attack upon the MMAR Schiff base by hydroxylamine during illumination. In addition, partial slowing down of the photocycle in these pigments, as observed for PR:MMAR 37, may contribute to this increased sensitivity to attack by hydroxylamine.

We could not detect any pigment formation with all‐*trans* phenyl retinal (PHE). The lack of a stable AR3:PHE pigment indicates that this analog cannot be stably contained in the retinal binding pocket of AR3, most probably due to the absence of methyl groups on the phenyl ring, which strongly contribute to correct positioning and stabilization in the retinal binding pocket 38, 39, 40, 41. This mirrors results obtained with GR, which does not appear to bind PHE, and is in contrast to PR, which forms a stable blueshifted PHE pigment 22. PR contains a F152 residue on helix E above the retinal *β*‐ionone ring, which presumably stabilizes the retinal in the binding pocket, while GR contains the small G178 which may not be sufficient for the formation of a stable PHE pigment (Fig. 2). However, in contrast to GR, AR3 contains a large tryptophan residue (W148) at the homologous position. Surprisingly, as well, BR, which has a set of residues lining the binding pocket identical to AR3 (Fig. 2, Table S1), was reported to slowly bind PHE, yielding a stable, blueshifted pigment, when expressed in its native environment 32, 42. Hence, we surmise that, upon expression in *E. coli*, AR3 can only bind its ligand while still in its folding stage. This would imply that PHE cannot attain stable binding at this stage and also would explain why we did not achieve regeneration of AR3 opsin postculture.

AR3 showed excellent regeneration with the locked ALL‐E analog. The absorbance band of AR3:ALL‐E did not shift significantly compared to that of AR3:A1 (Table 1), confirming data reported previously for other opsins 22, 31. This implies that retinal is bound in a 6‐s‐*trans* conformation in AR3 as well. This accounts for a portion of the spectral shift induced upon incorporation of A1 into the opsin binding pocket (opsin shift), since free A1 prefers the 6‐s‐*cis* conformation and the corresponding conformational twist into 6‐s‐*trans* already induces a redshift (Fig. S1) 31.

Retinal A2 caused a ~20 nm redshift in the absorbance bands of AR3 and AR3(F229S) relative to A1, with maximum absorbance at 578 and 581 nm, respectively, (Fig. 3 and Table I) corroborating previous findings with AR3 and other microbial rhodopsins 22, 25, 30, 43, 44, 45. The 14F analog also induced a redshift (ca 27 nm) in the absorbance band of AR3, accompanied by a strong broadening of the main absorbance band (Fig. S3). The 14‐Fluoro substitution was previously reported to redshift the absorbance band of BR by ~20 nm 46, and we also observed comparable redshifts with PR and GR (Fig. S3). Remarkably, however, incorporation of 3‐methoxy A2 (MOA2, Fig. 1) blueshifted the peak absorbance of AR3 and AR3(F229S) relative to A1, in striking contrast to PR and GR, for which we observed very large redshifts 23. MOA2 did however induce a very strong inhomogeneous broadening of the absorbance bands (Fig. 3), implying the presence of overlapping spectral components. This effect was also reported for PR and GR 23. On the other hand, incorporation of MMAR did result in a significant redshift for AR3 relative to A1 (36 nm to a *λ*max of 592 nm; Table 1 and Fig. 3), which is comparable to the redshifts of 48 and 30 nm reported for PR and GR, respectively 23. In addition, incorporation of MMAR in AR3 also induced a strong inhomogeneous broadening of the absorbance band (Fig. 3), but without the spectral wings observed in PR and GR extending to 850–900 nm 23 The mutation F234S in PR strongly enhances the lower energy spectral bands of PR:MMAR peaking at about 690 and 740 nm 23. In contrast, AR3(F229S):MMAR displayed a comparatively small 20 nm redshift relative to the corresponding A1 pigment (Table 1), but still showed a strong broadening of the main absorbance band (Fig. 3b).

**Table 1:** Spectral properties and proton‐pumping activity of the AR3 and AR3(F229S) pigments generated in this study

Pigment	Retinal analog	*λ*_max_ (nm)	Normalized proton‐pumping			
			H^+^ white	H^+^ 617	H^+^ 660	H^+^ 730
AR3 WT	A1	556	+++	++++	+++	**−**
	A2	578	+++	++++	+++	+
	MOA2	546	+	+	nd	nd
	MMAR	592	++	+++	+++	**++++**
	14F	583	nd	nd	nd	nd
	All‐E	562	nd	nd	nd	nd
AR3(F229S)	A1	560	**++++**	**++++**	++	**−**
	A2	581	+++	++++	**++++**	++
	MOA2	537	+	+	nd	nd
	MMAR	580	++	+++	+++	+++

The *λ*_max_ was determined from hydroxylamine difference spectra in solubilized membrane vesicles in 2.5% DDM. Accuracy of the *λ*_max_ values ±2 nm. The proton‐pumping activity was determined under four illumination conditions. To facilitate comparison, and more clearly show the general trend, all proton‐pumping rates of the pigments are normalized to the highest activity attained within a set of light conditions. For instance, AR3(F229S):A1 displayed the highest activity of 2 H^+^ mol^−1^ s^−1^ under white light illumination. Therefore, the proton‐pumping activity under white light illumination for all analog pigments was normalized to 2. The pigments with the highest activity attained for each set are the following: White light – AR3(F229S):A1 (2 H^+^ mol^−1^ s^−1^); 617 nm – AR3(F229S):A1 (1.4 H^+^ mol^−1^ s^−1^); 660 nm – AR3(F229S):A2 (1 H^+^ mol^−1^ s^−1^) and 730 nm – AR3:MMAR (0.3 H^+^ mol^−1^ s^−1^). For further details see the corresponding Materials and Methods section. Explanation of symbols: ++++: 100–70% of highest rate under that condition; +++: 70–40%; ++: 40–20%; +: 20–5%; **−**: <5%. nd = not determined.

### Fluorescence properties

AR3 is reported to have dim fluorescence emission, peaking at 687 nm, of which the intensity is sensitive to the transmembrane voltage 15, 34. Since the mutation D95N abolished the proton‐pumping ability of AR3, but retained voltage sensitivity and fluorescence emission with a greater brightness and dynamic range, it has been used as an optogenetic membrane potential sensor 15. Near‐IR resonance Raman spectroscopic measurements of AR3(D95N) indicate that at a molecular level the voltage sensing is mediated by the effects of the electric field on the protonation↔deprotonation equilibrium of the cytoplasmic accessible Schiff base 20, and this was corroborated by electrophysiological data 34. A combination of random and site‐saturation mutagenesis has been used to improve the fluorescence brightness of AR3 and AR3(D95N). Several variants were generated that show a 10‐ to 20‐fold increase in quantum yield along with a 40–50 nm redshift in peak emission 27, 47, 48. The AR3 variants termed Quasars, and their Archon derivatives display a redshifted emission band peaking around 720 nm 29, 48.

In order to assess the fluorescence properties of the AR3 and AR3(F229S) analog pigments generated in our study, the emission spectra of the A1, A2, MOA2 and MMAR pigments were recorded with excitation at their respective *λ*_max_ values (Fig. 3, bottom panels). The fluorescence properties of the F229S mutant and WT AR3 are very similar. All A1, A2 and MOA2 pigments showed weak emission bands peaking between 600 and 670 nm. In contrast, the MMAR pigments of AR3 and AR3(F229S) both showed a strong and significantly redshifted emission band peaking around 815 nm. This redshift is larger than that reported for the nonpumping AR3 variant containing a merocyanine retinal analog, which displayed an emission band peaking at 770 nm with an 8.5‐fold increase in brightness 28. We did not measure the quantum yield of the analog pigments in our study. However, we estimate that the brightness of the MMAR pigments is at least 10‐fold higher than that of WT AR3:A1, corroborating previous observations for the MMAR pigments of PR 24, 37.

### Proton‐pumping activity

**Figure 4:**
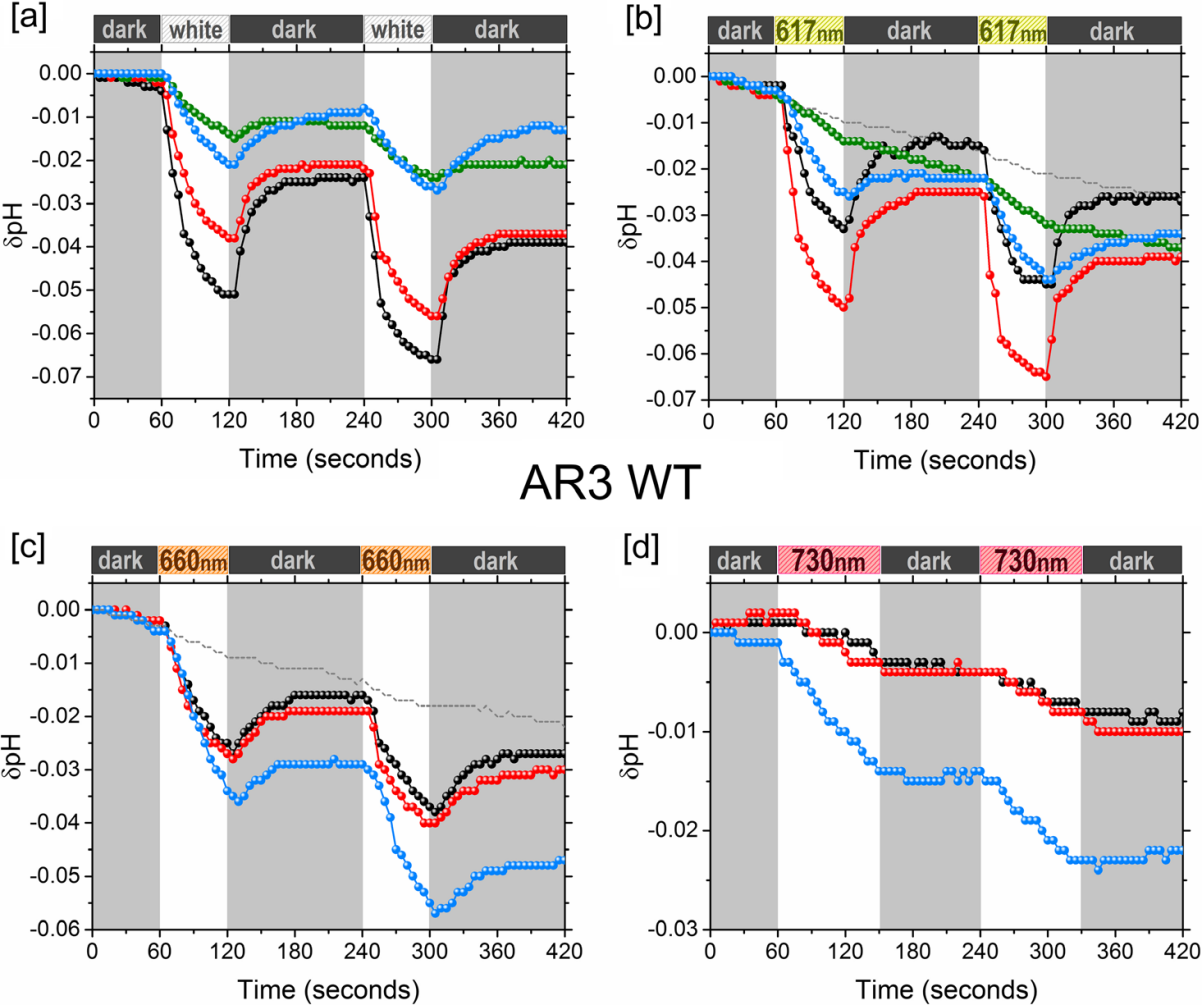
Proton‐pumping traces of AR3 under white light, 617, 660 and 730 nm LED illumination. A1 (black), A2 (red), MOA2 (green) and MMAR (blue). Examples of control experiments are represented by a dotted gray line. The drift in the controls is due to a small drift in the pH electrode. In panel d, the control overlaps with the black curve. Control experiments, illumination range and intensity of the light sources are detailed in the Supporting Information.

**Figure 5:**
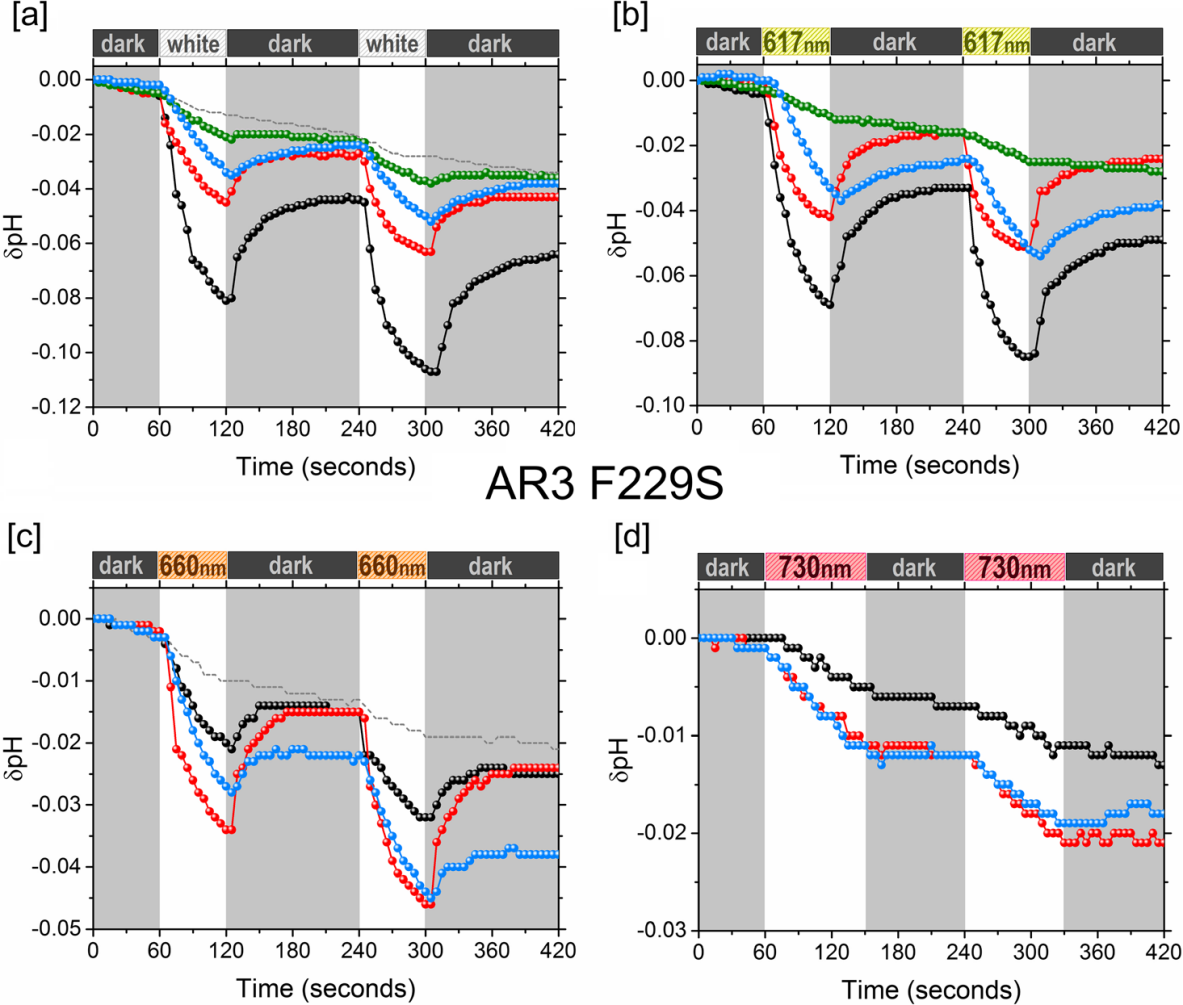
Proton‐pumping traces of AR3(F229S) under white light, 617, 660 and 730 nm LED illumination. A1 (black), A2 (red), MOA2 (green) and MMAR (blue). Examples of control experiments are represented by a dotted gray line. The drift in the controls is due to a small drift in the pH electrode. In panels b and d, the control overlaps with the green and black curve, respectively. Control experiments, illumination range and intensity of the light sources are detailed in the Supporting Information.

Proton‐pumping activity was assayed for the A1, A2, MOA2 and MMAR pigments of AR3 and AR3(F229S) using starved *E. coli* cell suspensions, and the proton‐pumping rates were estimated as described previously for the eubacterial rhodopsins 23. Several illumination conditions were applied using white light, 617, 660 and 730 nm LED lights (Figs. 4 and 5). The spectral range and intensities of the light sources are detailed in the Supporting Information. The relative pumping activity of the analog pigments under the various illumination conditions is presented in Table 1.

We observed that under white light illumination, the A1 pigment of AR3 showed a much lower pumping rate (ca 1.2 H^+^ mol^−1^ s^−1^) as compared to rates reported for the A1 pigments of PR and GR (ca 4 H^+^ mol^−1^ s^−1^ and 8 H^+^ mol^−1^ s^−1^, respectively, under the same conditions 23). This agrees with the up to five‐fold higher photocurrents generated by GR as compared to AR3 upon expression in oocytes 49. The activity of the AR3 analog pigments (Table 1) also was observed to be substantially lower than that reported for the same GR and PR analog pigments 22, 23.

In white light, AR3(F229S):A1 showed roughly a two‐fold increase in proton‐pumping rate compared to the WT AR3:A1 pigment. A2 pigments retained significant proton‐pumping activity in the range of 70–100% of the A1 activity for both AR3 and AR3(F229S). In contrast, the MOA2 and MMAR pigments showed much lower pumping rates under white light illumination, in the order of 15–30% of A1 activity. This trend is similar to what we observed for the analog pigments of PR and GR 23.

The reduction in pumping activity of the MMAR analog pigments will be at least partially due to their strong fluorescence emission, which dissipates part of the excitation energy. Considering this strong emission and the low pumping rate of the AR3:MMAR pigment, we surmise that in optogenetic applications, this pigment would function better as a NIR‐fluorescent voltage sensor than as a NIR‐active neuronal silencer. For the latter application, mutants with a higher pumping rate are required. The F229S mutant is a first step in that direction. The combination of retinal analogs with mutations therefore provides the exciting prospect of tuning the function of AR3 toward specific optogenetic and other photobiotechnological applications.

All A1, A2 and MMAR pigments retain significant activity under 617 LED illumination, while in contrast, the MOA2 pigments showed an almost complete loss of activity (Figs. 4 and 5, Table 1). This phenomenon is similar to previous observations with GR and PR where the proton‐pumping activity of MOA2 pigments was also strongly reduced under red light illumination 23. In correspondence with their absorbance profile, the A1 and A2 pigments show a marked decrease in activity under 660 and 730 LED illumination, relative to white light and 617 LED illumination. In fact, no activity can be seen for the A1 pigments at 730 LED illumination. Slight activity was noted for the A2 pigments with 730 LED illumination presumably due to their redshifted absorbance spectra relative to the A1 pigment. In contrast, when we normalize over LED photon flux, pigment concentration and absorbance cross section, we estimate that the pumping activity of the MMAR pigments remains more or less constant across the various illumination conditions. This corroborates our data with the MMAR pigments of PR and GR 23. This congruent pattern for members from different prokaryotic domains (AR3 from the archaea, PR from the eubacterial proteobacteria and GR from the eubacterial cyanobacteria) evokes the concept that the retinal analog MMAR would be able to induce NIR‐excitable activity in all prokaryotic rhodopsins.

### The retinal binding pocket environment

From this study, it is apparent that AR3 and GR share specific functional properties, despite the low sequence homology between these two microbial rhodopsins (~22% identity). The orthologous F→S mutation enhances expression of the resulting AR3 and GR pigments and increases their proton‐pumping activity, but has only a small impact on the absorbance band in the visible part of the spectrum (Table 1, Ref. 23). In contrast, in the case of PR, the orthologous F→S mutation (F234S) results in a 20 nm redshift with a strong reduction in proton‐pumping activity 23, 50. Furthermore, upon combination with MMAR, this F234S mutation induces a striking 180 nm redshift in the absorbance band in PR relative to PR:MMAR, while little shift was observed for the MMAR pigment of GR(F260S) relative to GR:MMAR 23 and in fact a small blueshift for AR3(F229S) (Table 1). We thereby decided to take a closer look at the retinal binding pocket of our homology models for AR3, PR and GR and compared it to the crystal structure of BR (PDB:3HAR) (Figs. 2 and Table S1, see Supporting Information). From this comparison, it is obvious that the protein residues lining the retinal binding pocket are entirely conserved between AR3 and BR (Table S1), which is in line with their high sequence homology (57% identity). In addition, BR and AR3 have almost identical resonance Raman spectra which reflects the nearly identical all‐*trans* chromophore structure and interaction with the binding pocket 20. On the other hand, the binding pocket of PR appears to deviate the most from AR3, especially for residues located near the *β*‐ionone ring and between C11‐C15 on the polyene chain of the retinylidene moiety. Such differences in the binding pocket environment will in turn alter the electronic distribution of the retinylidene moiety through modified electrostatic interactions.

Another likely consequence of the different binding pocket is alteration in the local hydrogen‐bonding network, presumably also involving bound water molecules 19, 51. The F234S mutation in PR is located in the vicinity of the Schiff base region 22, which may explain its potential to modulate the resonance pattern in MMAR in a different manner than for AR3 and GR 23, 24. The comparable spectral behavior of the F→S mutations in AR3 and GR may originate in the high sequence identity of the retinal binding pocket (ca 80%; Table S1). In this context, it is noteworthy that a congruent behavior of GR and AR3 was also observed for the fluorescent properties. Mutations that enhanced the emission of the GR:A1 combination, when applied to the orthologous positions in AR3, also enhanced the emission of the AR3:A1 combination 52. However, considering the low overall sequence identity, shared effects like the increase in expression and pumping activity need to be viewed from a larger perspective, in fact involving the full structure and dynamics of the protein. Such studies would provide further insight into the versatility of protein folding and dynamics.

**Figure 6:**
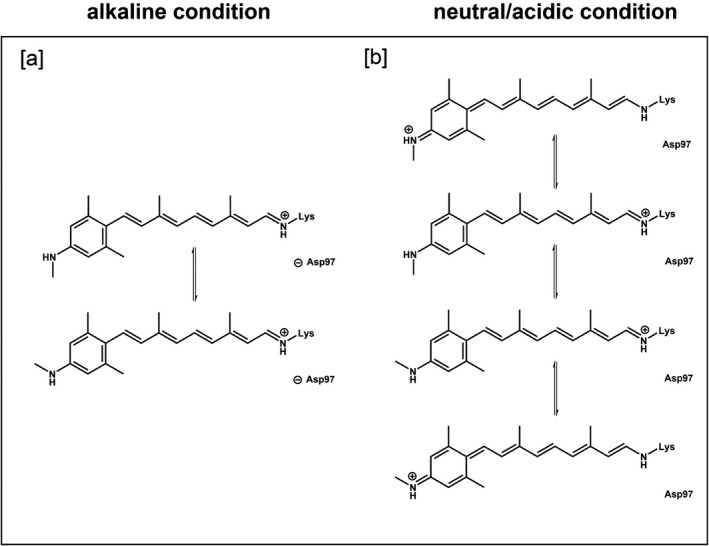
Resonance boundary structures hypothetically accessible to a MMAR chromophore. The F234S mutation in PR induces an effect similar to protonation of the counterion Asp97 23.

The most noteworthy difference between AR3 and GR on one hand and PR on the other hand is the effect of the orthologous F→S mutation on the spectral properties of the MMAR analog pigments. In AR3 and GR, this results in a small blue and red shift, respectively, with respect to the A1 pigments. However, in PR, this effectuates a very large shift into the NIR 23. Considering that the MMAR chromophore has access to resonance structures (Fig. 6), we propose that the sterical properties and tightness of the opsin binding site in the vicinity of the C3 position of the aromatic ring will be an important factor in determining the “resonance space” of the MMAR chromophore. We have shown before that the more voluminous dimethyl analog of MMAR (DMAR) has lower affinity for and lower stability in GR and AR3 as compared to PR 23, 24. This suggests that the binding sites of GR and AR3 are more restrictive around the aromatic ring of MMAR than that of PR. Consequently, we surmise that the larger rotational freedom and corresponding additional “resonance space” for the PR:MMAR chromophore will lead to broader and strongly redshifted absorbance bands, similar to what has been described for cyanine and azulenic dyes 53, 54, 55.

## Conclusion

We present several variants of the proton pump AR3 with novel spectral and functional properties and prospects. Most interestingly, our results unequivocally demonstrate that MMAR pigments of AR3 and its single mutant AR3(F229S) can be activated by near‐infrared light. This is in congruence with data reported for eubacterial proton pumps 23. Hence, chromophore modification using MMAR has the potential to become an effective strategy toward near‐infrared modulation of microbial rhodopsins. In addition, the AR3:MMAR pigments show a strong fluorescence emission band in the near‐infrared spectral region, which is roughly ten times brighter than that of the native pigment and is the most redshifted emission band reported for analog pigments of AR3. We anticipate that these NIR‐active pigments will have significant potential in a variety of biotechnological applications. For instance, in neuroscience, near‐infrared active optogenetic tools are highly desired due to the improved penetration of this light in mammalian tissue 28, 29, 56. Hence, the AR3:MMAR pigments generated in this study would be particularly suited for the generation of near‐infrared activable fluorescent voltage sensors. Further protein engineering to enhance their pump rate should also render them candidates for optogenetic stimulation of deep brain regions. Although most mammalian cells, including neurons, contain endogenous retinal A1, this usually is only present in very low (submicromolar) levels. It has been demonstrated that properly timed supplementation of HEK293 cells with 1–2 μm retinal analogs generates the corresponding analog pigments without significant effects on cell viability 28. In the case of MMAR analog pigments, the presence of low levels of A1 pigment would not interfere, since the A1 pigments are not activated by near‐infrared photons. Furthermore, very interesting applications can be envisioned in expanding the spectral window of oxophotosynthetic organisms 4, 57, of biomimicking photosynthetic tools 58 and in the artificial leaf concept 59, 60.

## Supporting information


**Appendix S1.** Primer Sequences.
**Figure S1.** Absorbance spectra of retinal analogs in organic solvents.
**Figure S2.** Normalized absorbance spectra of purified AR3:A1 (solid black) and AR3(F229S):A1 (dotted grey) pigments.
**Figure S3.** Normalized absorbance bands of the 14F analog pigments of AR3 (pink), PR (magenta) and GR (dotted pink).
**Figure S4.** Spectral intensity profile of the 730 nm LED used in the proton pumping experiments.
**Table S1.** Amino acid residues located in the retinal binding pocket of AR3, their corresponding residues in BR, PR and GR and their position relative to the retinylidene carbons.
