# Supplementary material for: Redshifted and Near‐infrared Active Analog Pigments Based upon Archaerhodopsin‐3
Source: Photochem Photobiol. 2019 Apr 8;95(4):959–68. doi: 10.1111/php.13093 (PMC6849744; doi:10.1111/php.13093)
Supplement: Supplementary file 1 — Appendix S1. Primer Sequences. Figure S1. Absorbance spectra of retinal analogs in organic solvents. Figure S2. Normalized absorbance spectra of purified AR3:A1 (solid black) and AR3(F229S):A1 (dotted grey) pigments. Figure S3. Normalized absorbance bands of the 14F analog pigments of AR3 (pink), PR (magenta) and GR (dotted pink). Figure S4. Spectral intensity profile of the 730 nm LED used in the proton pumping experiments. Table S1. Amino acid residues located in the retinal binding pocket of AR3, their corresponding residues in BR, PR and GR and their position relative to the retinylidene carbons. [file PHP-95-959-s001.pdf]

Supporting Information

Red-shifted and near-infrared active analog pigments based upon archaerhodopsin-3

Srividya Ganapathy*, Svenja Kratz, Que Chen, Klaas J. Hellingwerf, Huub J.M de Groot, Kenneth J. Rothschild, Willem J. de Grip*

*Corresponding authors

Primer Sequences

Primer name	Nucleotide sequence
AR3-F229S-fwd	5’-GGCTCAATCCTGTTGAGATCTCGGGCTATTCTG-3’
pet28-T7-rev	3’-TCAAGACCCGTTTAGAGGCCC -5
pet28-T7-fwd	5’-CGCTTTAATTATGCTGAGTGATATCCC -3’
AR3-F229S-rev	3-AGCCCGAGATCTCAACAGGATTGAGCCAAAGCC-‘5

The underlined nucleotides code for the mutation site

Proton pumping assay:

The cells were washed twice in starvation buffer (SB) containing 250 mM KCl, 10 mM NaCl, 10 mM $MgSO_4$, 100 µM $CaCl_2$, 10 mM Tris-HCl pH 7 and were starved by incubation with continuous mixing for 4 days at RT. The cells were washed another 3 times and resuspended in 5 ml SB supplemented with 40 µM final concentration of valinomycin. The cell suspension was incubated for 30 min in the dark at RT.

The following light sources with spectral range and intensity were used for the proton-pumping assay: white light (DLED9-T, DEDOLIGHT; 420-800 nm; 800 $\mu E.m^{-2}.s^{-1}$); 617 nm LED (M617L3, Thorlabs; 590-645 nm; 1500 $\mu E.m^{-2}.s^{-1}$); 660 nm LED (M660L4, Thorlabs; 610-700 nm; 800 $\mu E.m^{-2}.s^{-1}$); 730 nm LED (M730L4, Thorlabs; 665-775 nm; 160 $\mu E.m^{-2}.s^{-1}$). Light-induced pH changes were measured with a pH microelectrode (SenTix MIC, WTW) and the readout was monitored by a pH meter (Inolab pH 7310, WTW) connected to a computer. The following light regime was used: 1 min dark, 1 min light, 2 min dark, 1 min light, 2 min dark. For the 730 nm illumination of all species, a longer illumination period of 1.5 min was used. A 2 x concentrated cell suspension (cells from 50 ml culture in 4 ml starvation buffer) were used for 660nm and 730 nm illumination. Pumping rates were calculated using two independent trials. A calibration curve was measured using 0.1M HCl and 0.1M oxalic acid. Pumping rates were calculated as protons/sec from the initial rate of the light-induced pH change, if required corrected for baseline drift in controls (starved cells without expression of AR3-opsin or without retinal). Molecular pumping rates could subsequently be calculated after assay of the pigment concentration.

The 730 nm LED generates a low level (< 5 % of the total intensity) of deep red photons (< 700 nm; Fig S4). Considering the relatively low intensity of the 730 nm LED (see above), this small deep red fraction will not trigger significant pump activity. In fact, in case of MMAR pigments no significant difference in pump activity was measured with or without a Schott RG710 long-pass filter in front of the 730 nm LED. Hence, the MMAR pigments are truly near-infrared activable (> 700 nm).

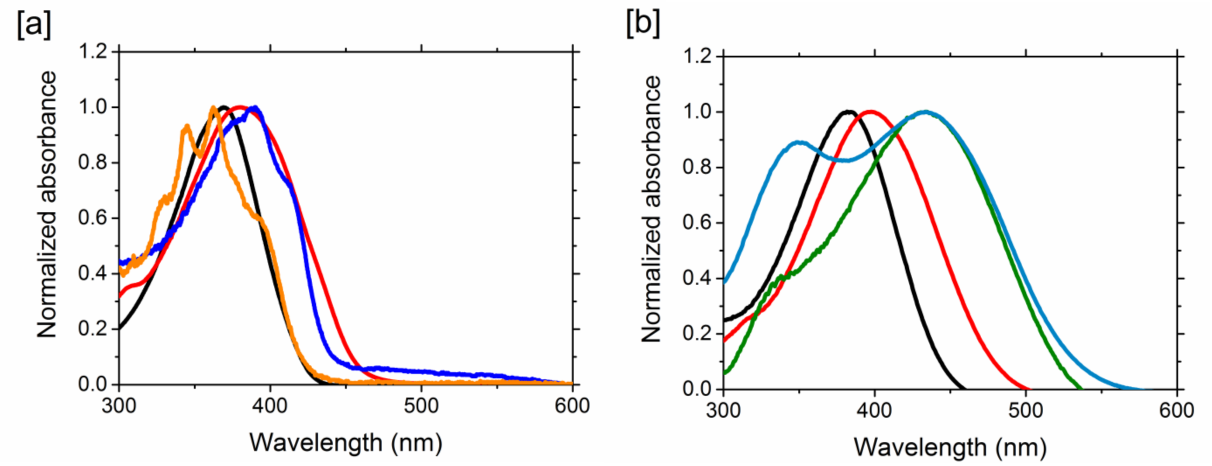

Figure S1: Absorbance spectra of retinal analogs in organic solvents. [a] all-*trans*-retinal (A1, black), all-*trans*-3,4-dehydroretinal (A2, red), all-*trans*-all-E-locked-retinal (ALL-E, blue); all-*trans*-1,2,3,4-didehydro-1,5-desmethyl-retinal (PHE, orange) using hexane as a solvent, with the exception of ALL-E (methanol) [b] A1 (black), A2 (red), all-*trans*-3-methoxy-3,4-dehydroretinal (MOA2, green) and all-*trans*-3-methylamino-16-nor-1,2,3,4-didehydroretinal (MMAR, blue) using dimethyl formamide as a solvent.

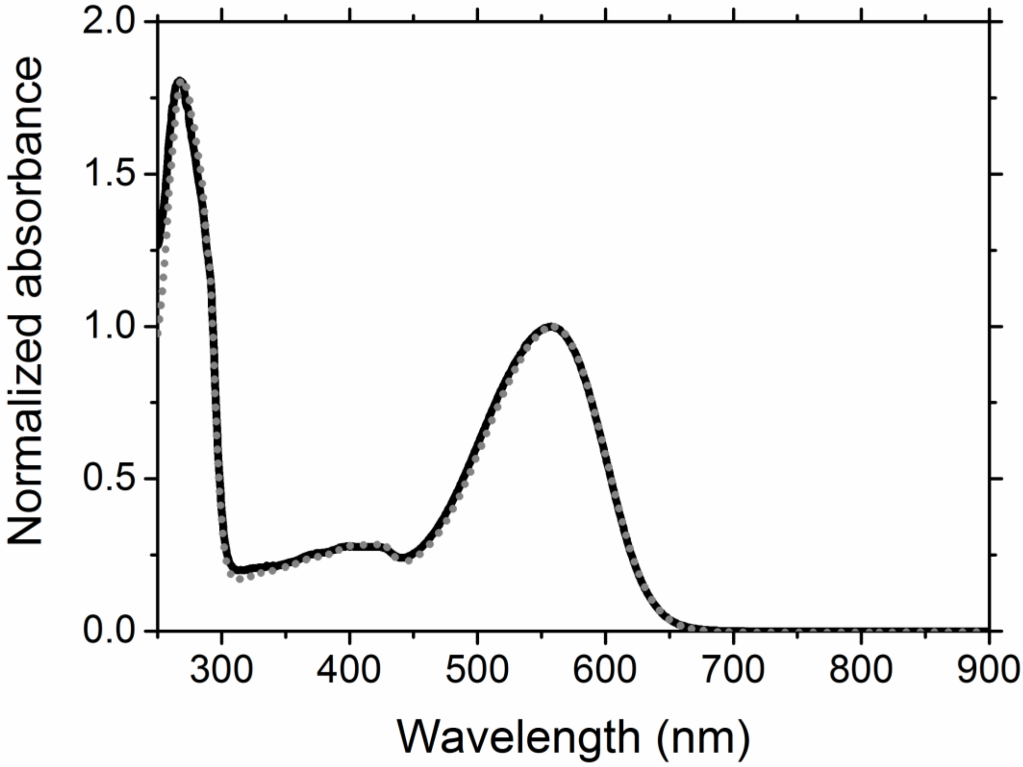

Figure S2: Normalized absorbance spectra of purified AR3:A1 (solid black) and AR3(F229S):A1 (dotted grey) pigments

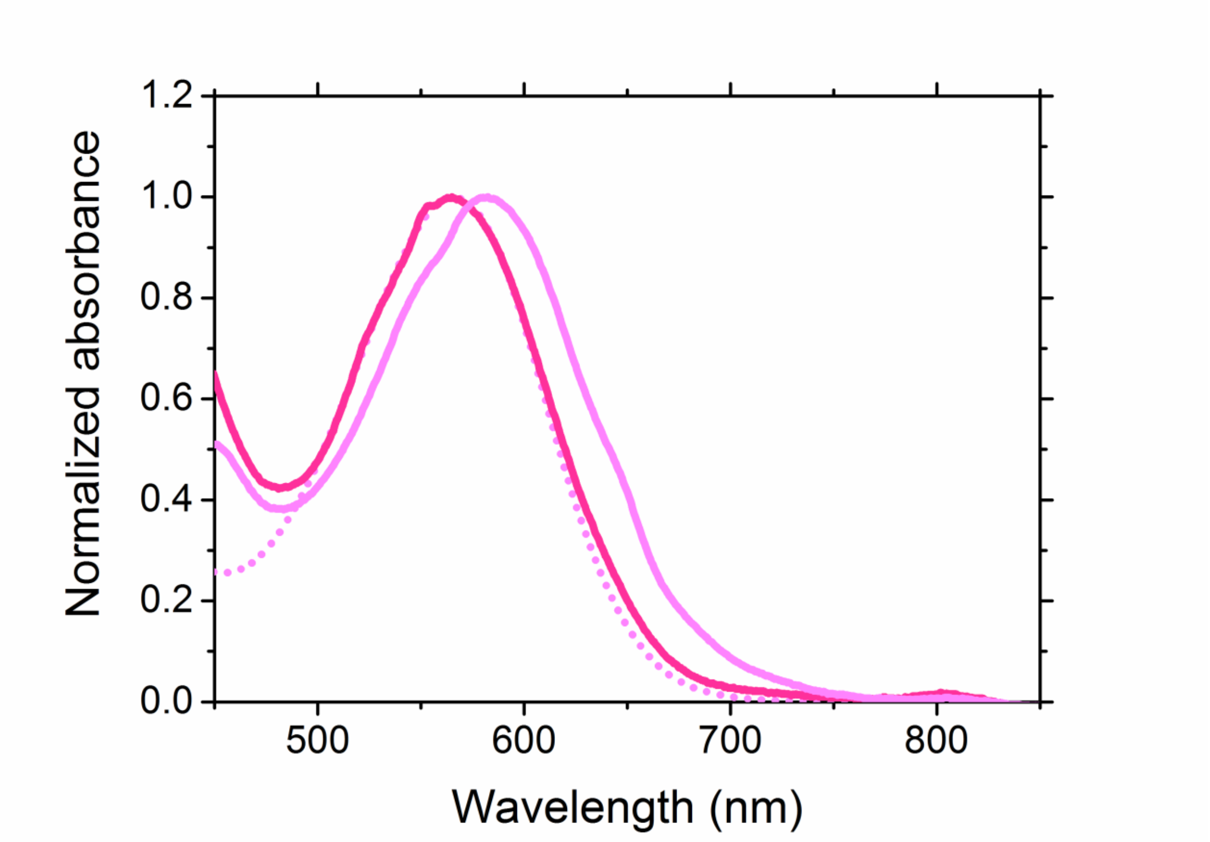

Figure S3: Normalized absorbance bands of of the 14F analog pigments of AR3 (pink), PR (magenta) and GR (dotted pink). The bands were obtained from difference spectra before and after treatment with hydroxylamine, as described in the Materials and Methods sections of the main text. The λ_{max} values of AR3:14F, PR:14F and GR:14F are 583nm, 565nm and 565 nm respectively, which reflects red-shifts of 27, 45 and 25 nm, respectively, with respect to the corresponding A1 pigments.

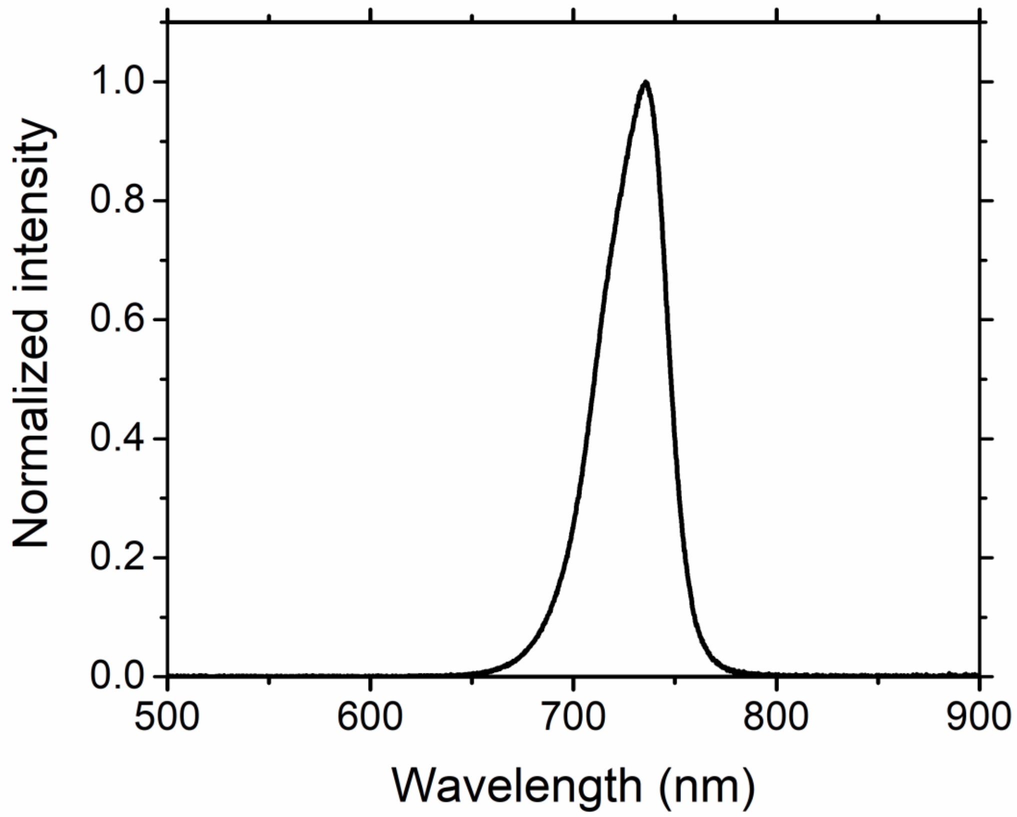

Figure S4: Spectral intensity profile of the 730 nm LED used in the proton pumping experiments.

**Located near retinal carbon /s**	**AR3**	**BR**	**PR**	**GR**
C1, C16, C17	Y93	Y83	Y95	Y119
C15	D95	D85	D97	D121
C15	T99	T89	T101	T125
C13, C20	T100	T90	V102	V126
C13, C20	L103	L93	L105	L129
C5-7, C18	M128	M118	M134	M158
C8, C9, C19	I129	I119	I135	I159
C2-4	W148	W138	F152	G178
C5, C18	S151	S141	G155	S181
C4, C5	T152	T142	C156	T182
C5, C18	M155	M145	W159	F185
C10-13, C20	W192	W182	W197	W222
C10-13	Y195	Y185	Y200	Y225
C3-5, C18	P196	P186	P201	P226
C1-4, C16, C17	W199	W189	Y204	Y229
C15	D222	D212	D227	D253
C15	A225	A215	N230	A256
C15	K226	K216	K231	K257

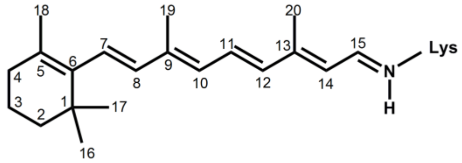

Table S1: Amino acid residues located in the retinal binding pocket of AR3, their corresponding residues in BR, PR and GR and their position relative to the retinylidene carbons. All conserved residues are in black, while residues which deviate from AR3 are highlighted in red (PR) and green (GR). Residues are included that range within 4 Å from the nearest retinylidene carbon atom.
